# Agreement and Concordance Regarding Reproductive Intentions and Contraception Between Husbands and Wives in Rural Ballabgarh, India

**DOI:** 10.4103/0970-0218.62548

**Published:** 2010-01

**Authors:** Kapil Yadav, Bir Singh, Kiran Goswami

**Affiliations:** Centre for Community Medicine, All India Institute of Medical Sciences, New Delhi, India

**Keywords:** Agreement, family planning, husbands and wives, rural

## Abstract

**Background:**

Traditionally, women have been the chief respondents in most demographic and health surveys focusing on family planning; the role of men has been limited. However, in recent years there has been realization of the importance of men's role in family planning.

**Aims and Objectives:**

To assess the levels of agreement and concordance between husbands and wives regarding reproductive intentions and contraception.

**Materials and Methods:**

A cross-sectional survey was carried out in 200 randomly selected married couples (in the age range of 15-44 years) in village Dayalpur, Haryana. Data pertaining to reproductive intentions and contraception was collected and the level of agreement (kappa statistics) between husbands and wives was calculated.

**Results:**

The observed concordance was 67.5% for ideal family size, 84.5% for contraceptive attitude, 88.5% for fertility desire, 93.5% for unmet need, and 97% for report of number of currently living children. The adjusted kappa statistic varied from a low of 0.43 (*P* ≤ 0.001) (ideal family size) to a high of 0.96 (*P* ≤ 0.001) (number of living children) with contraceptive attitude (0.7) (*P* ≤ 0.001), unmet need (0.88) (*P* ≤ 0.001), and current use of contraception (0.93) (*P* ≤ 0.001) having kappa values in between. Overall, a greater degree of agreement was observed for reproductive health events as compared to family planning attitudes and intentions. An in-depth analysis of the responses in the current study provides further evidence of male domination in decision making.

**Conclusion:**

In surveys pertaining to reproductive health events, the wife's response can be taken as proxy for the couple's response, but for assessing family planning attitudes and intentions, there is a need to collect information from husbands and wives separately.

## Introduction

Traditionally, women have been the respondents in most knowledge, attitude, and practice surveys related to family planning, contraceptive prevalence surveys, and demographic and health surveys; the role of men has been limited to providing information only on household or demographic characteristics and to granting permission for interviewing the women.(1–3) Demographers imitated contraceptive developers in their focus on women, so that fertility rates and measures of contraceptive prevalence, unwanted fertility, and unmet demand for contraception were all based on women's reports.

Demographers and program managers now realize that programs focused exclusively on either men or women may fail in their purpose because most sexual, family planning, and childbearing decisions are made or may potentially (and perhaps ideally) be made by both partners together. The International Conference on Population and Development (ICPD) document recognizes the couple as a unit by referring frequently to ‘couples and individuals’ and further states that ‘the aim of family planning programmes must be to enable couples and individuals to decide freely and responsibly on the number and spacing of their children….’(4) Available studies show that in many developing countries males often dominate when important decisions are taken in the family, such as on reproduction, family size, and contraceptive use.(5)

All the above mentioned facts favor the interviewing of both husbands and wives when ascertaining family planning attitudes. But the additional resources and efforts necessary for collecting information from both husbands and wives counterbalance the gains of this approach. The current study was designed to assess the levels of agreement and concordance between husbands and wives regarding family planning. If low concordance is observed then the former approach would be advisable, whereas in case there is high agreement between husbands and wives then interviewing wife alone might suffice.

## Materials and Methods

The current study was conducted in village Dayalpur in Haryana, India. The said village is approximately 40 km from NCR New Delhi and is one of 28 villages covered by the Comprehensive Rural Health Services Project (CHRSP), a collaborative project between the Center for Community Medicine, All India Institute Medical Sciences, and Haryana (State) Government. It comprises a secondary care hospital and two primary health centers (PHCs). The rural population under the project is approximately 80,000, that of Dayalpur village alone being 8,000. PHC Dayalpur is staffed by three doctors, four trainee doctors, and support staff (the manpower is in excess of the prescribed norms for India as the PHC is directly managed by a medical college). Domiciliary preventive and promotive health care is provided by health workers, both male and female, in a planned way, such that every household is visited at least once every fortnight by one of them.

A unique feature of CRHSP is the management information system (MIS), which enables maintenance of a computerized record of every individual. Every child born in the CRHSP is allotted a unique number and all relevant information pertaining to an individual is recorded in a longitudinal manner. The MIS enables health workers to generate a monthly computerized work plan for improving the quality of health care delivery and also helps in better monitoring of service.

The target population for the study included all married couples residing in Dayalpur village wherein the wife was in the 15-44 years age-group. Out of 1136 eligible couples, 200 were selected randomly. The eligible couple was list generated by the MIS from the CRHSP records and simple random sampling was performed using the random number table. This study was part of the postgraduate dissertation of the first author and no additional resources were allocated for it.

This is a cross-sectional study carried out over the period July 2003-June 2004. Data were collected using a pretested semistructured interview schedule, which was administered by the first author himself. The interviews were conducted at the respondents' homes and both husbands and wives were interviewed on same day, but separately to prevent contamination. Data entry was made using EpiInfo 2002 and analysis was done using Microsoft Excel (Widows Office 2000) and SPSS (version 10 for Windows). Proportion, mean, median, range, and standard deviation were the descriptive statistics used. Level of agreement between husbands and wives was analyzed using kappa statistics. As observed by Banikole *et al*. ‘…finding an adequate measure for distinguishing between spousal concordance that occurs by chance and actual agreement is a methodological difficulty….’(6) Ted Byrt *et al*. observed that the ‘…kappa statistic is influenced by the distributions of data across the categories that are used (prevalence).’ As kappa statistics may be influenced by the presence of more than two categories and by high prevalence of the outcome under consideration, we used weighted kappa and prevalence-adjusted kappa wherever appropriate.(7)

## Results

The mean age of husbands enrolled in the study was 33.4 (SD:7.3 years) and that of wives was 28.5 (SD: 6.5 years). The mean duration of schooling for husbands was 10.1 years (SD: 4 years) and for wives it was 6.5 years (SD:5.1 years). Compared to 32.5% of wives, only 9% husbands reported never attending school. Caste analysis of the couples revealed almost equal distribution of upper castes (mainly Rajputs and Brahmins; 36%), backward castes (Jat, Nai, etc.; 33%), and scheduled castes (31%). Socioeconomic status was assessed using the Udai-Parikh scale.(8) The majority of the couples belonged to the lower middle class (56.5%), followed by middle class (35%), lower class (4.5%), upper middle class (3.5%), and upper class (0.5%).

The mean age at cohabitation was 22.6 years (SD: 3.5 years) for males and 17.5 years (SD: 3.2 years) for females. The age at marriage was 12-32 years for males and 7-27 years for females. Amongst the males, 24.5% were married before the age of 21 years, whereas 42.5% of females were married before the age of 18 years (i.e., before the legal minimum age for marriage as per the Child Marriage Restraint Act of 1978).(9)

Table 1 shows cross-tabulation of the number of living children as reported by husbands and wives. The diagonal of the table represents the axis of agreement between husbands and wives. Overall in 97% (95% CI:93.5 to 98.8%) of cases, both partners reported the same number of living children and the kappa statistic was 0.96 (*P* ≤ 0.001), which corresponds to excellent agreement.(10) In all six cases where there was difference in reported number of children, it was the husband who reported lesser number of children. Interestingly enough, all the under-reported children were females. It appears that men tend to under-report the number of living daughters–probably they count only sons as ‘children.’

**Table 1:** Agreement between husbands and wives regarding number of living children

Parity	Wives					Total
		
	0	1	2	3	4	
Husbands						
0	23 (11.5)	2 (1)	1 (0.5)	-	-	26 (13)
1	-	32 (16)	-	-	-	32 (16)
2	-	-	63 (31.5)	1 (0.5)	1 (0.5)	65 (32.5)
3	-	-	-	65 (32.5)	1 (0.5)	66 (33)
4	-	-	-	-	11 (5.5)	11 (5.5)
Total	23 (11.5)	34 (17)	64 (32)	66 (33)	13 (6.5)	200 (100)

Figures in parenthesis are in percentages

Cross-tabulation for ever use of contraception (irrespective of the method) showed 95% (95% CI: 90.9 to 97.5%) agreement between husbands and wives [Table 2]. The kappa statistic was 0.86 (*P* ≤ 0.001), which corresponds to excellent agreement according to Fleiss' classification.(9) Ever use of contraception was reported by only two (1%) wives when husbands reported that they had never used any contraception. In both the cases, the wives had taken oral pills and it could be that they did not inform their husbands regarding this. Ever use of contraception was reported by eight (4%) husbands when wives reported otherwise. In all eight cases, the method used by the husbands was a male-dependent method: Abstinence and withdrawal in three cases each and condoms in two cases. This difference may be attributable to differences in perception between spouses regarding traditional methods or may be indicative of existence of multiple sexual partners (condom use).

**Table 2:** Agreement between husbands and wives regarding contraceptive practice, attitude, and fertility desire

Ever use of contraception	Wives		Total
		
	No	Yes	
Husbands			
No	42 (21)	2 (1)	44 (22)
Yes	8 (4)	148 (74)	156 (78)
Total	50 (25)	150 (75)	200 (100)
Current use of contraception			
Husbands			
No	65 (32.5)	-	65 (32.5)
Yes	6 (3)	129 (64.5)	135 (67.5)
Total	71 (35.5)	129 (64.5)	200 (100)

**Contraceptive attitude**	**Disapprove**	**Approve**	**Total**

Disapprove	6 (3)	18 (9)	24 (12)
Approve	13 (6.5)	163 (81.5)	176 (88)
Total	19 (9.5)	181 (90.5)	200 (100)

**Fertility desire**	**Wives desire for children**		**Total**
		
	**Want no more**	**Want more**	

Husbands desire for children			
Want no more	115 (57.5)	12 (6)	127 (63)
Want more	11 (5.5)	62 (31)	73 (36.5)
Total	126 (63)	74 (37)	200 (200)

Figures in parenthesis are in percentages

Current use of contraception as reported by husbands and wives showed 97% (95% CI: 93.5 to 98.8%) overall agreement [Table 2]. The kappa statistic was 0.93 (*P* ≤ 0.001), which corresponds to excellent agreement. Only in six cases was disagreement observed, and in all of them current use of contraception was reported by the husband while the wife reported using none. The methods reported by husbands were again observed to be male-dependent methods: Abstinence in four cases and withdrawal in two cases. It may be inferred that the disagreement could be due to differences in perception regarding traditional methods amongst spouses as well as hesitation on the part of females to report use of traditional methods like withdrawal.

Table 2 shows that there is 84.5% (95% CI:78.7 to 89.2%) agreement between husbands and wives in attitude toward contraception; both partners approved of contraception in 81.5% cases. The unadjusted kappa statistic was 0.19 (*P* ≤ 0.001). We used prevalence- and bias-adjusted kappa for parameters for which prevalence was high. The prevalence-adjusted kappa for contraceptive approval for husbands and wives was 0.7 (*P* ≤ 0.001), which corresponds to excellent agreement. In 9% of cases, wives approved of contraception whereas husbands did not, and in 6.5% of cases, husbands approved of contraception whereas wives did not.

Cross-tabulation for fertility desire showed agreement between husbands and wives in 88.5% (95% CI: 83.2 to 92.5%) of cases, 57.5% of couples wanting no more children and 31% wanting more children [Table 2]. The unadjusted kappa statistic was 0.75 (*P* ≤ 0.001) and the prevalence-adjusted kappa was 0.78 (*P* ≤ 0.001). The disagreement was evenly divided across remaining two cells, with 6% cases where the wife desired more children while the husband did not and 5.5% cases where the husband desired more children but wives did not. Cross-tabulation of ideal family size showed that in 67.5% (95% CI: 60.5 to 73.9%) of cases there was agreement between husband and wife [Table 3]. In 15.5% of cases, the husband wanted more children than the wife did, while in 17% of cases the wife wanted more children than the husband did. The unadjusted kappa statistic was 0.38 (*P* ≤ 0.001) and weighted kappa, adjusting for more than two possible outcomes, was 0.43 (*P* ≤ 0.001).

**Table 3:** Agreement between husbands and wives regarding ideal family size

Ideal family size	Wives-desired no. of children			Total
		
	1	2	3	
Husbands-desired no. of children				
1	27 (13.5)	24 (12)	1 (0.5)	52 (26)
2	12 (6)	95 (47.5)	9 (4.5)	116 (58)
3	1 (0.5)	18 (9)	13 (6.5)	32 (16)
Total	40 (20)	137 (68.5)	23 (11.5)	200 (100)

Figures in parenthesis are in percentages

Cross-tabulation of unmet need of contraception revealed concordance amongst husbands and wives in 93.5% (95% CI:89.1 to 96.4%) of cases [Table 4]. Unmet need in both was reported by 82.5% and none by 6.5%. The unadjusted kappa statistic was 0.73 (*P* ≤ 0.001) and prevalence-adjusted kappa was 0.88 (*P* ≤ 0.001). In all cases where disagreement was seen (6.5%), it was the wife who reported unmet need whereas the husband reported none. There was no case where the husband reported an unmet need but the wife did not. This finding possibly indirectly reflects the predominant role of husbands in decision making regarding use of contraception. Probably, even when the woman does not want to have any more children, they are not free to use contraception without the husband's consent. Perhaps wives are unable to communicate their need for contraception to husbands or to convince them, whereas the husbands are always able to do so.

**Table 4:** Agreement between husbands and wives regarding unmet need for family planning

Ucnmet need for family planning	Wives		Total
		
	No	Yes	
Husbands				
No	165 (82.5)	13 (6.5)	165 (82.5)
Yes	0 (0)	22 (11)	22 (11)
Total	178 (89)	35 (17.5)	200 (100)

Figures in parenthesis are in percentages

## Discussion

In our study, the observed agreement between husbands and wives regarding reporting of reproductive health events and family planning attitudes and intentions varied from a low of 67.5% (95% CI:60.5 to 73.9%) for ideal family size to a high of 97% (95% CI:93.5 to 98.8%) for number of currently living children. Similarly, the adjusted kappa statistic varied from 0.43 (*P* ≤ 0.001) for ideal family size to 0.96 (*P* ≤ 0.001) for number of living children. In any study on current reproductive attitude and behavior amongst monogamous couples with both partners in their first marriage (which was the case in the present study) only one valid response can be recorded for a couple for objective events like number of children born and current use of contraception. Any differences indicate response error on the part of one spouse or both. Determining which respondent (or if either) gave the correct response is usually impossible because validation studies of these indices are lacking. The minor differences in objective events in the present study can be explained by the male-dominated culture of this study population.

A high level of agreement in number of living children was also reported by Vlassoff *et al*. in a study from rural Maharashtra, where only 0.4% disagreement was observed for number of currently living sons and daughters. They also concluded that women reported live births more accurately than men.(11) A study from Turkey reported that ‘spousal reports on most fertility and contraceptive use measures demonstrated moderate to high concordance, whereas reports of approval of family planning showed only fair concordance.’(12) In a study using data from six demographic and health surveys of sub-Saharan Africa, contraceptive use agreement ranged from 47% to 82%, but among couples in whom one or both reported use, the ‘both’ category represented less than half in all nations except Zimbabwe.(13) Whereas discrepancies in the reporting of events indicate reporting errors on the part of one or both spouses, differences of attitudes and intentions are expected because these are subjective indicators. The in-depth analysis of responses to contraceptive attitude, fertility desire, unmet need, and family size in the current study provides further evidence for male domination in decision making. This effect is similar to that reported by numerous other studies globally. Available studies show that in many developing countries, males often dominate in decision making in the family, including in issues related to reproduction, family size, and contraceptive use. Research in Kenya suggests that contraception is 2-3 times more likely to be used when husbands, rather than wives, want to cease childbearing.(14) Male involvement not only helps in making a contraceptive more acceptable, but also makes its effective use and continuation more likely.(15–17) On the other hand, even if the wife wants to use a contraceptive, she may not be able to use it or may be forced to discontinue the method if the husband disapproves of contraception. In a study conducted in Indonesia, husbands' approval was the most important determinant of contraceptive use.(18)

The concept of unmet need of family planning describes those currently married individuals who are not using any method of contraception but who do not want any more children or want to wait for at least 2 years before having another child; pregnant women whose pregnancies were mistimed or unwanted and non-menstruating women whose last births were mistimed or unwanted are also included in the definition.(19) The significant difference in unmet need of husbands and wives, combined with the dominant role played by husbands, indicates that current estimates of unmet need based on wives' perceptions alone may be overestimates. Thus, there is need for formulating policies keeping the husbands' perspectives also in view. A few researchers have already questioned the validity of the estimates of unmet need derived from information collected only from women.(20,21) Taking into consideration male intentions allows a clearer understanding of why family planning programs have not been more successful in developing countries.

This study was part of a postgraduate thesis and thus was primarily exploratory in nature. Our sample size was restricted owing to limitations of time and logistics. All interviews were conducted by the author himself. The fact that interviews were conducted by a male researcher may question the validity of responses, especially that of the wives; however, every effort was made to offset this by establishing good rapport with the respondents. The researcher was accompanied by a female worker belonging to the community itself to help in this. Moreover, by introducing himself as a doctor he hopefully dispelled some of the apprehensions of the respondents. The fact that a single researcher conducted all the interviews may have led to overall better standardization. The findings of this study are applicable to Dayalpur village only and may not be generalizable to other villages owing to differences in socioeconomic environments.

There is need to further study the extent to which spouses influence each other's contraceptive attitudes in order to derive a precise measure of the power relationship between spouses with respect to reproductive behavior. This will address the theoretical question posed by all agreement studies: Does agreement between partners or their reporting of similar reproductive preferences mean equal input by both partners to reproductive decision making or simply demonstrate complete domination of one over the other?

## Conclusion

Overall, a greater degree of agreement was observed for reproductive health events as compared to family planning attitudes and intentions. The latter are more subjective outcomes and can be expected to vary among spouses. Thus, we can infer that for reproductive health events, wives' responses can be taken as proxy for the couple's response but for family planning attitudes and intentions there is a need to collect information both from husbands and wives, especially so in a patriarchal society like India.
